# Haplotype analysis of *SERPINE1* gene: Risk for aneurysmal subarachnoid hemorrhage and clinical outcomes

**DOI:** 10.1002/mgg3.737

**Published:** 2019-07-03

**Authors:** Mingkuan Lin, Christoph J. Griessenauer, Robert M. Starke, R. Shane Tubbs, Mohammadali M. Shoja, Paul M. Foreman, Nilesh A. Vyas, Beverly C. Walters, Mark R. Harrigan, Philipp Hendrix, Winfield S. Fisher, Jean‐Francois Pittet, Mali Mathru, Robert H. Lipsky

**Affiliations:** ^1^ Department of Systems Biology George Mason University Fairfax Virginia; ^2^ Department of Neuroscience INOVA Health System Fairfax Virginia; ^3^ Department of Neurosurgery Geisinger, Danville Pennsylvania; ^4^ Research Institute of Neurointervention, Paracelsus Medical University Salzurg Austria; ^5^ Department of Neurosurgery and Radiology University of Miami Miami Florida; ^6^ Children’s of Alabama Birmingham Alabama; ^7^ Department of Neurosurgery University of Alabama at Birmingham Alabama Alabama; ^8^ Department of Neurosurgery Saarland University Medical Center, Saarland University Homburg Germany

**Keywords:** clinical vasospasm, Delayed Cerebral Ischemia, *SERPINE1*, Subarachnoid Hemorrhage, tissue plasminogen activator

## Abstract

**Background:**

Aneurysmal subarachnoid hemorrhage (aSAH) has high fatality and permanent disability rates due to the severe damage to brain cells and inflammation. The *SERPINE1* gene that encodes PAI‐1 for the regulation of tissue plasminogen activator is considered an important therapeutic target for aSAH.

**Methods:**

Six SNPs in the *SERPINE1* gene (in order of rs2227631, rs1799889, rs6092, rs6090, rs2227684, rs7242) were investigated. Blood samples were genotyped with Taqman genotyping assays and pyrosequencing. The experiment‐wide statistically significant threshold for single marker analysis was set at *p* < 0.01 after evaluation of independent markers. Haplotype analysis was performed in Haplo.stats package with permutation tests. Bonferroni correction for multiple comparison in dominant, additive, and recessive model was applied.

**Results:**

A total of 146 aSAH patients and 49 control subjects were involved in this study. The rs2227631 G allele is significant (*p* = 0.01) for aSAH compared to control. In aSAH group, haplotype analysis showed that G5GGGT homozygotes in recessive model were associated with delayed cerebral ischemia (*p* < 0.01, Odds Ratio = 5.14, 95% CI = 1.45–18.18), clinical vasospasm (*p* = 0.01, Odds Ratio = 4.58, 95% CI = 1.30–16.13), and longer intensive care unit stay (*p* = 0.01). By contrast, the G5GGAG carriers were associated with less incidence of cerebral edema (*p* < 0.01) and higher Glasgow Coma Scale (*p* < 0.01). The A4GGGT carriers were associated with less incidence of severe hypertension (>140/90) (*p* < 0.01).

**Conclusion:**

The results suggested an important regulatory role of the *SERPINE1* gene polymorphism in clinical outcomes of aSAH.

## 1 INTRODUCTION

Aneurysmal subarachnoid hemorrhage (aSAH) accounts for 75%–80% of nontraumatic SAH and has high fatality and permanent disability rates due to the severe damage to brain cells and inflammation (Priebe, 2007). The common risk factors for aSAH included hypertension, smoking, and patterns of alcohol consumption (Larrew et al., 2015). Nevertheless, aSAH is also considered as the common end point of the interaction of environmental, biomechanical, cellular, molecular, and genetic processes that underlie the formation and rupture of cerebral aneurysms. While triggers for aSAH remain poorly understood, mounting evidence suggested that genetic factors not only contribute to aneurysm formation, but also to aneurysm rupture (Ladner, Zuckerman, & Mocco, 2013; Theodotou et al., 2017). A better understanding of the genetic influence on aneurysm formation and rupture risk may aid clinicians in the identification of patients at higher risk for aSAH.

The *SERPINE1* gene (OMIM# 173360) that encoded Plasminogen activator inhibitor‐1 (PAI‐1) regulates the function of tissue cell plasminogen activator (tPA) and urokinase plasminogen activator (uPA). Current studies indicated that extracellular matrix remodel, atherosclerosis, and fibrinolytic dysfunction were considered as important pathogenic mechanisms of cerebral aneurysm (Chalouhi et al., 2012; Steucke, Tracy, Hald, Hall, & Alford, 2015; Tang, McKenna, & Rovit, 1973). While tPA/uPA plays a pivotal role in the homeostasis of blood coagulation/fibrinolysis and extracellular matrix regulation (Hu et al., 2008; Lu, Takai, Weaver, & Werb, 2011), the *SERPINE1* gene that regulate tPA/uPA level could be a therapeutic target for aSAH. The increased tPA and decreased PAI‐1 level were also observed in subarachnoid hemorrhage patients (Ji, Meng, & Wang, 2014). In this study, a total of six single nucleotide polymorphisms (SNP) in the human *SERPINE1* gene were investigated for their association to aSAH. Rs2227631 (−884 A > G) and rs1799889 are located in the promotor region. Rs6092 (Thr15Ala) and rs6090 (Ile17Val) are missense variants located in Exon 2. Rs2227684 is intronic while rs7242 is located in the 3' untranslated region. Meta‐analysis showed that the rs2227631 polymorphism was significantly associated with the risk of coronary artery disease and the rs1799889 polymorphism was significantly associated with the risk of myocardial infarction and cerebral infarction (Liu et al., 2018). The rs6092 SNP has been reported to be associated with abdominal visceral fat in postmenopausal women (Bouchard, Mauriège, Vohl, Bouchard, & Pérusse, 2005) and susceptibility of osteonecrosis among children with acute lymphoblastic leukemia (French et al., 2008). Additionally, the rs7242 G allele was associated with an increased risk of myocardial infarction in nonsmoker populations and with increased serum insulin levels in myocardial infarction patients (Morange et al., 2007).

Clinical vasospasm (CV) and delayed cerebral ischemia (DCI) are important complications after SAH. DCI is the leading cause of mortality and morbidity of patients after aSAH (Rowland, Hadjipavlou, Kelly, Westbrook, & Pattinson, 2012). DCI is traditionally thought as outcome caused by vasospasm, although, the pathophysiological etiology of DCI is currently believed to be multifactorial (Budohoski et al., 2014; Geraghty & Testai, 2017). Other factors excepting vasospasm, include cerebral vascular dysregulation, microthrombosis, cortical spreading depolarizations, and neuroinflammation (Geraghty & Testai, 2017). To assess the pathological role of the *SERPINE1* gene and aSAH, as well as CV and DCI, the association between the phenotypic conditions and other clinical measures and *SERPINE1* gene polymorphism was conducted in a case–control study. The linkage disequilibrium in *SERPINE1* polymorphisms was examined and haplotype analysis performed with outcome measures in patient and control groups.

## 2 METHODS

### 2.1 Patient population

Peripheral blood samples of 195 subjects (146 aSAH patients and 49 controls) who were enrolled in the Cerebral Aneurysm Renin Angiotensin System (CARAS) study were used for genetic evaluation. Subjects or their legal representative provided informed written consent prior to enrollment according to the Institutional Review Board‐approved protocol for the two clinical sites (University of Alabama at Birmingham and Inova Health System, USA). Methods have been previously described for the CARAS study (Foreman et al., 2016, 2017), but a brief review follows. The decision of aSAH was established through the admission CT scan or xanthochromia of cerebral spinal fluid (CSF). Aneurysmal hemorrhage was confirmed by CT angiography (CTA) or DSA. Exclusion criteria were subjects under 19 years old, with any associated genetic predisposition that could contribute to cerebral aneurysm formation, with any systemic diseases (congestive heart failure or cirrhosis) that could interfere with renin‐angiotensin system activity, or with abnormalities of the cerebral vasculature. The control group was composed of trauma patients (age > 19) without known genetic risk factors for cerebral aneurysm formation and with unremarkable head and neck CTA (no cerebral aneurysm or other vascular malformation). Both aSAH patients and controls were enrolled within 72 hr of admission. Patients were treated in accordance with guidelines for the management of aSAH, including intensive care unit (ICU) monitoring, treatment of hydrocephalus, early (<48‐hr) intervention for aneurysm treatment, oral nimodipine, maintenance of euvolemia, and surveillance for clinical vasospasm and DCI. Among the 146 aSAH patients, 34 suffered CV and 31 were diagnosed with DCI, and 25 were suffered both CV and DCI. The raw data supporting the conclusions of this manuscript will be made available by the authors, without undue reservation, to any qualified researcher.

### 2.2 Definition of Clinical Vasospasm and Delayed Cerebral Ischemia

Definitions of CV and DCI were detailed in a previous study (Hendrix et al., 2017). Briefly, CV was defined as a new focal or global neurological deficit, or deterioration of at least 2 points on the Glasgow Coma Scale (GCS), not explained by other confounding clinical processes. Angiographic vasospasm was defined as arterial narrowing on CTA or DSA not due to atherosclerosis, catheter‐induced vasospasm, or vessel hypoplasia. In addition, vasospasm was diagnosed with transcranial Doppler ultrasonography (TCD) findings of a mean systolic middle cerebral artery (MCA) pressure of more than 120 cm/sec with a Lindegaard ratio more than 3. CTA, DSA, and TCD were obtained at the discretion of the treating neurosurgeon.

The diagnosis of CV was adjudicated by consensus of the study team and treated with hyperdynamic therapy as the first line of treatment. Patients with clinical vasospasm refractory to medical treatment were treated in the endovascular suite at the discretion of the treating neurosurgeon. CT or MRI was routinely performed when the patient was transferred from the ICU to the ward. DCI was defined as low‐density areas on CT that corresponded to a vascular distribution, or MRI demonstrating a hyperintense area on a diffusion‐weighted imaging sequence with a corresponding hypointense apparent diffusion coefficient sequence correlate that corresponded with a vascular territory. Images were evaluated locally and clinicians were blinded to the genetic analysis.

### 2.3 Outcome Measures

Outcome measures included CV, DCI, and functional outcomes at the time of discharge from the acute hospital setting, and at last follow‐up using the Modified Rankin Scale (MRS) (0 = full recovery; 6 = death). All outcome data were obtained blinded to the results of the genetic analysis. Functional outcome was assessed either in clinic or via telephone interview with the patient or with a surrogate if the patient was unable to participate.

### 2.4 Laboratory and Statistical Genetic Evaluation

Genomic DNA was isolated from whole blood using a Gentra Puregene DNA extraction kit according to the manufacturer's specifications (QIAGEN Inc., USA). Four SNPs in *SERPINE1* gene (rs2227631, rs6090, rs2227684, and rs7242) were genotyped by 5' exonuclease (TaqMan) allelic discrimination assays. Commercial TaqMan assays were designed and performed according to the vendor (Thermo Fisher Scientific, Inc. USA). SNPs rs1799889 SNP and rs6092 were genotyped by a sequence by synthesis method (Pyrosequencing) according to vendor's instruction on a Pyromark Q24 platform (QIAGEN Inc.). The forward and reverse primer used for SNP rs1799889 polymerase chain reaction (PCR) were 5'–ACTTACACGTTGGTCTCTCCTGTTT–3' and 5'–CCAACAGAGGACTCTTGGTCTT–3'. The sequencing primer used for rs1799889 was 5'–GATACACGGCTGACTCCC–3'. The forward and reverse primer for SNP rs6092 PCR were 5'–GTTGCAGGAAACAAGAAGAGCAG–3' and 5'–Biotin–ATAGGGTGAGAAAACCACGTTGC–3'. The sequencing primer used for rs6092 was 5'–CCTGCCTAGTCCTGGGCC–3'. The genotype completion rate was 100% for each of the six SNPs evaluated in this study. Those samples testing with inconsistent or unsuccessful genotyping results were repeated until agreements were reached. Repeated experiments were performed in 10% random samples, and the agreements were 100%.

Statistical analysis was carried out in R statistical environment (version 3.4.4). Categorical variables were analyzed with chi‐square test. To prevent inflation of type I error caused by multiple comparisons and also to take the dependence between markers into consideration, the effective number of independent markers was examined using the Genetic Type I error calculator (GEC) (Li, Yeung, Cherny, & Sham, 2012). The results indicated that 4.5 independent markers (effective ratio = 0.75) for multiple testing correction. Accordingly, the experiment‐wide statistically significant threshold for single marker analysis was set at *p* < 0.011. Logistic regression involving other factors measured at patient administration, treatments and SNPs were conducted with significant level set at *p* < 0.05.

Haplotypes for each individual were carried out with an expectation maximization algorithm using the Haplo.stats (Schaid, Rowland, Tines, Jacobson, & Poland, 2002) package to infer the haplotype frequency in cases and controls, and to evaluate the association with outcome measures using embedded score function. The analyses were further performed by 10,000 permutation tests on phenotype data to obtain the statistical distribution and reduce the uncertainty of the P value. The significance level of these analyses was set at *p* = 0.05 after 10,000 permutation tests. In the permutation tests procedure, the score statistics were re‐calculated from the permutated re‐ordering of traits and covariates and the original ordering of genotype matrix. All haplotype association analyses in this study were adjusted with covariates for age, gender, and ethnic group. Only haplotype frequencies over 5% were included in the analysis. To correct for the inflation of type I error due to the comparison of haplotype‐based analyses, a Bonferroni correction was applied by taking the total number of haplotype blocks and haplotypes into consideration (Zhang, Calabrese, Nordborg, & Sun, 2002). For example, the corrected *p* value < 0.009 (*n* = 6, dominant model with 4 haplotypes and 2 blocks) and 0.02 (*n* = 3, recessive model with 2 haplotypes and 1 blocks) was regarded as significant. The visualization and calculation of linkage disequilibrium (LD) was conducted with LDMAP package (Kuo, Lau, & Collins, 2007).

## 3 RESULTS

### 3.1 Demographics

The mean age (*p* = 0.17) and sex distribution (*p* = 0.24) between aSAH and control groups were similar. Regarding the aneurysmal size, 92 (63.0%) patients had aneurysms < 7 mm, 47 (32.2%) had 7–12 mm, 6 (4.1%) had 13–24 mm, and the last 1 (0.7%) had giant aneurysm > 25 mm. The race (*p* = 0.05) distribution between aSAH and control is marginally significantly different, with higher African‐American in aSAH group than in control group. The data presented in Table 1 show the distribution of *SERPINE1* genotypes stratified by ethnic population in aSAH group. The rs2227631 and rs1799889 variants had marginal significance for Hardy–Weinberg Equilibrium (HWE) in African Americans (*p* = 0.07 and 0.02, respectively) (Table 1) and this also indicated in the higher frequency of rs2227631 G allele and rs1799889 5G allele frequency for aSAH patients than controls in Table 2 (*p* = 0.01 and *p* = 0.06, respectively). This is due to higher proportion of African Americans (40%) in the aSAH group compared to the population distribution of individuals of African descent (14% as of 2017) in the United States. Higher frequency of aSAH in African Americans has also been reported by others (Broderick, Brott, Tomsick, Huster, & Miller, 1992; Rosen et al., 2005).

**Table 1:** Genotype distribution stratified by race for aSAH patients

SNPID	Allele (1/2)	Group	Genotype			
			1/1	1/2	2/2	HWE P (χ^2^)
rs2227631 (−884 A > G)	A/G	European American	28	45	14	0.56
		African American	1	27	31	0.07
rs1799889 (−675 4G/5G)	4G/5G	European American	27	45	15	0.61
		African American	0	27	32	0.02
rs6092 (Thr15Ala)	A/G	European American	0	18	69	0.28
		African American	0	2	57	0.89
rs6090 (Ile17Val)	A/G	European American	0	1	86	0.95
		African American	0	6	53	0.68
rs2227684	A/G	European American	18	42	27	0.82
		African American	9	31	19	0.53
rs7242	T/G	European American	26	42	19	0.79
		African American	18	32	9	0.40

**Table 2:** Genotype and allele comparisons for *SERPINE1* gene polymorphisms for aSAH patients (*n* = 146) and control (*n* = 49)

SNPID	Allele (1/2)	Group	Genotype				P (χ^2^) (group)	Allele		
			1/1	1/2	2/2	HWE P(χ^2^)		1	2	P (χ^2^) (group)
rs2227631 (−884 A > G)	A/G	aSAH	29	72	45	0.98	0.04	130	162	0.01
		Control	18	22	9	0.62		58	40	
rs1799889 (−675 4G/5G)	4G/5G	aSAH	27	72	47	0.95	0.16	126	166	0.06
		Control	15	23	11	0.70		53	45	
rs6092 (Thr15Ala)	A/G	aSAH	0	20	126	0.37	0.15	20	272	0.17
		Control	0	3	46	0.83		3	95	
rs6090 (Ile17Val)	A/G	aSAH	0	7	139	0.77	0.40	7	285	0.40
		Control	0	1	48	0.94		1	97	
rs2227684	A/G	aSAH	27	73	46	0.83	0.41	127	165	0.19
		Control	12	26	11	0.66		50	48	
rs7242	T/G	aSAH	43	74	27	0.63	0.32	160	128	0.14
		Control	10	26	13	0.65		46	52	

### 3.2 Single SNP analysis and association of aSAH with other factors

The genotype and allele distributions for each of the six *SERPINE1* gene SNPs for the aSAH and control groups are shown in Table 2. None of the genotype frequencies showed significant deviation from HWE. The chi‐square analysis of single locus effects revealed that rs2227631 had a significant association with aSAH when analyzed by allele frequency (*p* = 0.01) and a trend using genotype frequency (*p* = 0.04). For rs2227631, when compared with AA homozygotes, the G allele carriers were represented more frequently in the aSAH group (Odds Ratio = 2.34, 95% confidence interval (CI) = 1.15–4.76, *p* = 0.02). Follow‐up logistic regression and chi‐square analysis showed that individuals with rs2227631 G allele (*p* = 0.02), and older age (*p* = 0.04) are associated with aSAH subjects more than controls. Individuals with severe hypertension (over 140/90) (*p* = 0.02) and ventriculoperitoneal shunt (VPS) treatment (*p* = 0.04) were associated with increased incidence of both CV and DCI.

### 3.3 Haplotype analysis

**Figure 1:**
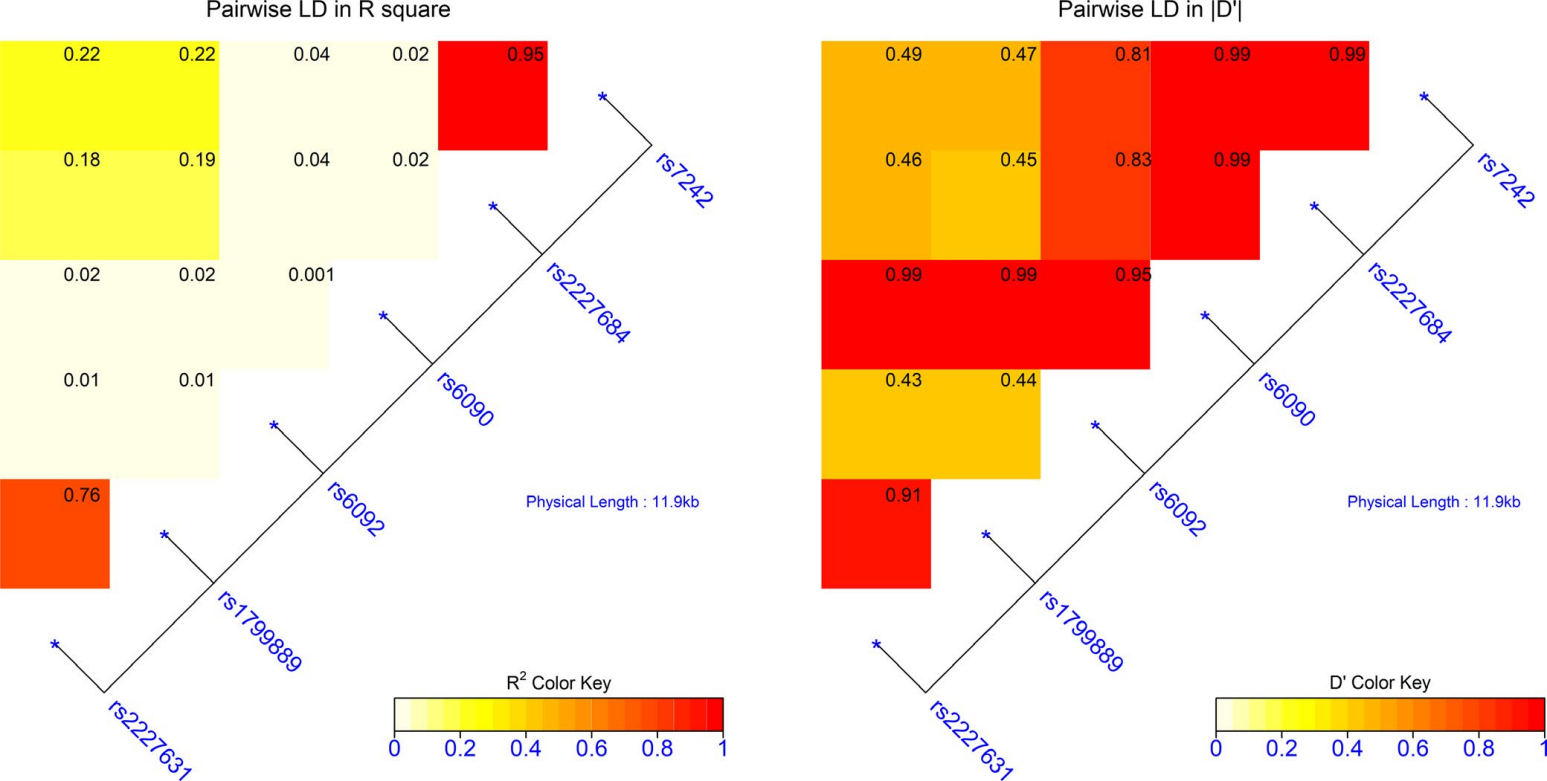
LD map showing block structure across the *SERPINE1* gene. Heat map color keys indicate the degree of R^2^ and D' and the number in each block indicate the calculated R^2^ and D'. The LD map is constructed with LDMAP package in R statistical environment

The association of the *SERPINE1* polymorphisms with aSAH was further investigated by looking into the distribution of *SERPINE1* haplotypes (Figure 1). All haplotype association analyses in this study were adjusted with covariates age, sex, and ethnic group. The negative and positive sign for the score function of haplotype analysis indicated the direction of association. In our data, the variables with binary properties were coded 0 for “without symptom” and coded 1 for “with symptom”. Thus, the negative sign indicated no association with symptom and positive sign indicated association with symptom. For other ordinal or quantitative variables, negative sign indicated association with lower scale and positive sign indicated association with higher scale. The structure of the *SERPINE1* haplotype could be divided into two blocks based on pairwise LD D′ ≥ 0.8 (Figure 1). The first block constructed with rs2227631 and rs1799889 of the six markers were in strong LD to each other and were both located in the promotor region of *SERPINE1*. For this two‐marker haplotype, there was an excess of G‐5G haplotype carriers in aSAH subjects relative to controls (Odds Ratio 0.57, 95% CI 0.35–0.92, *p* = 0.02)(Table 3A, detail in Table S1). However, the two‐marker haplotype was not significant in score function tests against incidence of aSAH, DCI, and CV. Cerebral Edema is associated with A‐4G carriers in aSAH subjects than G‐5G carriers (Odds ratio 3.01, 95% CI 1.60–5.66, *p* < 0.001).

**Table 3:** Haplotype analysis on aSAH outcomes

**(A) Block1 (rs2227631‐rs1799889)**								
Haplotype (Frequency)					Permutation P value			
**aSAH**	Case	Control	P (χ^2^)		Dominant	Additive		Recessive
A4 (0.44)	120	51	0.02		0.85	0.75		0.76
G5 (0.50)	157	38			0.17	0.36		0.97
Global					0.38	0.78		0.94
**Cerebral Edema**	Yes	No	P (χ^2^)		Dominant	Additive		Recessive
A4(0.41)	36	83	0.02		0.41	0.17		0.17
G5(0.53)	28	125			0.53	0.20		0.15
Global					0.65	0.38		0.19
**(B) Block2 (rs6092‐rs6090‐rs2227684‐rs7242)**								
**Hypertension (>140/90)**	Yes	NO	P (χ^2^)		Dominant	Additive		Recessive
AGGT(0.06)	13	6	0.44		0.98	0.98		
GGAG(0.42)	99	34			0.02(score = 2.36)	0.15		0.83
GGGT(0.47)	91	44			0.90	0.12		0.01(score = −2.48)
Global					0.09	0.48		0.03
	Permutation P value							
**Fisher CT Scale**	Dominant			Additive			Recessive	
AGGT(0.06)	0.34			0.35				
GGAG(0.42)	0.05 (score = −2.00)			0.16			0.89	
GGGT(0.47)	0.18			0.02(score = 2.34)			0.01(score = 2.45)	
Global	0.12			0.14			0.05	
**Last F/U MRS**	Dominant			Additive			Recessive	
AGGT(0.06)	0.22			0.23				
GGAG(0.42)	0.04 (score = −2.01)			0.01 (score = −2.69)			0.02 (score = −2.40)	
GGGT(0.47)	0.01 (score = 2.54)			0.02 (score = 2.38)			0.26	
Global	0.03			0.04			0.05	
**Glasgow Coma Scale**	Dominant			Additive			Recessive	
AGGT(0.06)	0.84			0.83				
GGAG(0.42)	0.10			0.05(score = 2.00)			0.11	
GGGT(0.47)	0.12			0.06 (score = −1.90)			0.14	
Global	0.27			0.24			0.16	
**(C) Block3 (rs2227631‐rs1799889‐rs6092‐rs6090‐rs2227684‐rs7242)**								
**Hypertension (>140/90)**	Yes	NO	P (χ^2^)		Dominant	Additive		Recessive
A4GGGT(0.10)	10	13	0.02		0.001(score= −3.19)	0.004(score = −2.80)		
G5GGAG(0.13)	27	7			0.04(score = 2.02)	0.19		
A4GGAG(0.29)	65	24			0.26	0.40		0.98
G5GGGT(0.32)	73	27			0.62	0.77		0.81
Global					0.007	0.06		0.97
**Cerebral Edema**	Yes	No	P (χ^2^)		Dominant	Additive		Recessive
A4GGGT(0.10)	10	13	0.003		0.03(score = 2.23)	0.01(score = 2.50)		
G5GGAG(0.13)	1	32			0.006(score = −2.77)	0.007(score = −2.68)		
A4GGAG(0.29)	24	64			0.96	0.75		0.50
G5GGGT(0.32)	21	77			0.82	0.70		0.61
Global					0.04	0.02		0.73
**DCI**	DCI	No DCI	P (χ^2^)		Dominant	Additive		Recessive
A4GGGT(0.10)	6	16	0.22		0.85	0.85		
G5GGAG(0.13)	3	30			0.33	0.28		
A4GGAG(0.29)	22	66			0.72	0.83		0.36
G5GGGT(0.32)	25	72			0.56	0.09		0.005(score = 2.75)
Global					0.85	0.27		0.008
**CV**	CV	No CV	P (χ^2^)		Dominant	Additive		Recessive
A4GGGT(0.10)	4	19	0.14		0.33	0.29		
G5GGAG(0.13)	3	31			0.12	0.11		
A4GGAG(0.29)	24	65			0.93	0.59		0.31
G5GGGT(0.32)	26	74			0.85	0.18		0.01(score = 2.54)
Global					0.34	0.21		0.01
	Permutation *p* value							
**Hutt and Hess scale**	Dominant			Additive			Recessive	
A4GGGT(0.10)	0.56			0.52				
G5GGAG(0.13)	0.01(score = −2.66)			0.02(score =−2.47)				
A4GGAG(0.29)	0.73			0.78			0.99	
G5GGGT(0.32)	0.58			0.30			0.20	
Global	0.05			0.06			0.42	
**Hospital Stay**	Dominant			Additive			Recessive	
A4GGGT(0.10)	0.80			0.66				
G5GGAG(0.13)	0.15			0.13				
A4GGAG(0.29)	0.76			0.94			0.72	
G5GGGT(0.32)	0.35			0.05(score = 1.91)			0.02(score = 2.53)	
Global	0.55			0.25			0.05	
**ICU Stay**	Dominant			Additive			Recessive	
A4GGGT(0.10)	0.83			0.70				
G5GGAG(0.13)	0.04(score = −1.98)			0.04(score = −1.96)				
A4GGAG(0.29)	0.78			0.86			0.93	
G5GGGT(0.32)	0.38			0.06(score = 1.93)			0.01(score = 2.64)	
Global	0.24			0.11			0.03	
**Last F/U MRS**	Dominant			Additive			Recessive	
A4GGGT(0.10)	0.48			0.27				
G5GGAG(0.13)	0.07(score = −1.80)			0.06(score = −1.89)				
A4GGAG(0.29)	0.09(score = −1.66)			0.07(score = −1.83)			0.26	
G5GGGT(0.32)	0.16			0.04(score = 2.05)			0.04(score = 2.11)	
Global	0.15			0.09			0.08	
**Glasgow Coma Scale**	Dominant			Additive			Recessive	
A4GGGT(0.10)	0.98			0.92				
G5GGAG(0.13)	0.002(score = 2.97)			0.002(score = 3.030)				
A4GGAG(0.29)	0.56			0.72			0.84	
G5GGGT(0.32)	0.41			0.18			0.13	
Global	0.03			0.02			0.31	

The second haplotype block was constructed with rs6092, rs6090, rs2227684, and rs7242. The GGAG carriers were associated with better outcomes after aSAH, such as lower last follow up MRS (additive and recessive model, *p* < 0.02) and higher GCS (additive model, *p* = 0.05)(Table 3B, detail in Table S2). The GGAG carriers were also associated with higher incidence of sever hypertension (>140/90) in dominant model for aSAH subjects (*p* = 0.02). By contrast, the GGGT carriers were associated with worse outcomes of aSAH, such as higher Fisher CT scale, higher last follow‐up MRS and lower GCS. The GGGT homozygotes were not associated with severe hypertension (>140/90) (*p* = 0.01) for aSAH subjects in recessive model. These results were marginally significant or not significant after multiple comparison correction. The haplotypes constructed with the four SNPs in the second block were not associated with aSAH, DCI, or CV (Table S2).

After combing the six SNP markers in the two blocks, the overall distribution of haplotypes was significantly different for DCI and CV in the recessive model (Global *p* = 0.01 for DCI and *p* = 0.02 for CV) (Table 3C, detail in Table S3). More specifically, the G5GGGT (32% haplotype frequency) homozygotes were associated with DCI (recessive model *p* = 0.005, Odds Ratio = 5.14, 95% CI = 1.45–18.18) and CV (recessive model *p* = 0.01, Odds Ratio = 4.58, 95% CI = 1.30–16.13). The G5GGGT homozygotes were also associated with worse outcomes after aSAH, such as longer ICU stay (*p* = 0.01), longer hospital stay (*p* = 0.02), and higher MRS (*p* = 0.04). By contrast, the G5GGAG carriers were associated with better outcomes, such as less incidence of cerebral edema (dominant and additive model *p* < 0.01), lower Hutt and Hess scale (dominant and additive model, *p* < 0.02) and higher GCS (dominant and additive model, *p* < 0.01). The aSAH subjects with A4GGGT carriers were associated with less incidence of severe hypertension (>140/90) (dominant and additive model, *p* < 0.01) but were associated with higher incidence of cerebral edema (dominant and additive model, *p* < 0.03). These results were marginally significant or not significant after multiple comparison correction.

## 4 DISCUSSION

### 4.1 Population stratification in African Americans for rs2227631 and rs1799889

Family studies have suggested a role of genetic factors in susceptibility to aSAH, but the underlying genetic risk factors remain poorly defined (Bor et al., 2008). This study investigated six SNPs in *SERPINE1* gene to evaluate the association of *SERPINE1* gene to the clinical outcomes of aSAH. The analyses have been adjusted for age, gender, and ethnic groups, which are the external factors that may impact *SERPINE1* gene expression (Cesari, Pahor, & Incalzi, 2010). In this study, we identified that the rs2227631 and the rs1799889 are in high LD and also found that G‐5G haplotype carriers have higher incidence of aSAH in African Americans. This result coincided with previous studies that shown higher incidence of aSAH in this population (Broderick et al., 1992; Rosen et al., 2005), and displayed a potential population stratification of rs2227631 G allele and rs1799889 5G allele for aSAH in African Americans. Further study with more aSAH subjects is necessary to confirm the suggested population stratification.

### 4.2 G5GGGT homozygotes are associated with worse aSAH outcomes

One major finding of this study was that G5GGGT homozygotes were associated with DCI, CV, and worse aSAH clinical outcomes. The common rs1799889 five G tract (5G allele) allows a transcriptional repressor protein to bind, reducing the level of PAI‐1 (Eriksson, Kallin, van't Hooft, Båvenholm, & Hamsten, 1995). Conversely, the four G tract (4G) allele increases PAI‐I transcription and is associated with increased circulating PAI‐1 levels (Kathiresan et al., 2005). This G5GGGT haplotype thus could implicate a decrease in PAI‐1 levels with a concomitant increase in tPA. DCI is the most important cause of mortality and poor neurological outcome after aSAH. The formation of DCI is multifactorial and one of the factors is cortical spreading depression (CSD) (Leng, Fink, & Iadecola, 2011). CSD is a self‐propagating wave triggered by multiple stimuli and travels at about 2–5 mm/min, which induced depolarization in neuron and glial and results in a redistribution of ions and neurotransmitters. This effect causes an increase in extracellular potassium ions and glutamate, leading to a depolarization cycle that ends in neuronal inactivation (Somjen, 2001). This is observed electrocorticographically as a period of transient depression in cortical electrical activity. CSD alters blood‐brain barrier (BBB) permeability by activating brain matrix metalloproteinase 9 (MMP‐9) (Gursoy‐Ozdemir et al., 2004). The increased MMP‐9 concentration in CSF after aSAH is a suggested biomarker for DCI (Triglia et al., 2016) and the elevated MMP‐9 after cerebral ischemia is associated with accelerated matrix degradation, disruption of the blood‐brain barrier, increasing the infarct size, and relating to hemorrhagic transformation (Dong, Song, Liu, & Guo, 2009; Turner & Sharp, 2016). In addition, studies showed that tPA is released after depolarization by calcium‐mediated mechanisms in neurons (Gualandris, Jones, Strickland, & Tsirka, 1996; Tsuji et al., 2005; Turner & Sharp, 2016), and tPA can upregulate MMP‐9 and other MMP subtypes (Tsuji et al., 2005; Wang et al., 2003). Thus, the G5GGGT haplotype that may associate with lower levels of PAI‐1, higher tPA, and higher MMP‐9 levels leading to increased risk of DCI and CV after aSAH via a CSD/tPA/MMP‐9‐mediated mechanism. The G5GGAG carriers associated with better aSAH outcome suggested the importance of rs2227684 or rs7242 to alter this mechanism. The result also coincided with our previous finding that the rs2227684 AA and rs7242 GG predicted a more favorable outcome after aSAH (Hendrix et al., 2017).

### 4.3 Interaction of SNPs to impact the aSAH clinical outcomes

Our results showed that the interaction of rs1799889 and rs7242 could impact the clinical outcomes of aSAH. The cerebral edema is associated with A4GGGT carriers, while G5GGAG carriers were associated better aSAH outcomes. In addition, A4GGGT carriers with aSAH were also associated with less incidence of severe hypertension. Current theory suggested that increased tPA level exacerbated brain edema and leaded to the worsening of neurological outcomes in intracerebral hemorrhage (Thiex et al., 2003; Thiex, Mayfrank, Rohde, Gilsbach, & Tsirka, 2004). One possible explanation is that tPA disrupted the BBB and extracellular matrix through binding of low‐density lipoprotein receptor‐related protein (LRP) signaling (Wang et al., 2003) or through tPA upregulated MMP‐9 protein (Lo, Wang, & Cuzner, 2002). But, the 4G allele that was associated with increased PAI‐1 levels and reduced tPA levels alone cannot explain the results. One plausible mechanism may involve the rs7242 SNP in the *SERPINE1* 3' untranslated region, which is a region known to be a target for RNA binding proteins or microRNA(miRNA)‐mediated translational and transcriptional instability (Fang & Rajewsky, 2011). By using bioinformatic tool MicroSNiPer (Barenboim, Zoltick, Guo, & Weinberger, 2010), we found that there are more putative miRNAs bind to the G allele than to the T allele (Table S4). When search from the RNA binding protein database (RBPmap) (Paz, Kosti, Ares, Cline, & Mandel‐Gutfreund, 2014), a putative allele‐specific RNA binding protein was discovered (Table S4), suggesting that the rs7242 G allele could be more likely bind to miRNA or a RNA binding protein than the T allele, although, the fate or functional alteration of mRNA after binding is dynamic (Hentze, Castello, Schwarzl, & Preiss, 2018). Nevertheless, the Ecto‐NOX disulfide‐thiol exchanger 1 (*ENOX1*) gene could have allele‐specific binding at rs7242. *ENOX1* is associated with vasculogenesis and angiogenesis in a zebrafish model (Venkateswaran et al., 2014). In that model, suppression of Enox1 protein impaired vasculogenesis and angiogenesis. Since the rs7242 G allele was associated with increased risk of myocardial infarction, the regulatory mechanisms between Enox1 and PAI‐1 needs further investigation. In addition to *SERPINE1*, other genes such as *PPARG*, *ARNTL*, and *SLC12A9‐ACHE* may also impact the level of PAI‐1 and hence tPA levels (Huang et al., 2012).

### 4.4 Study limitations

One limitation of this study is that the proportion of African Americans in all participants (about 34%), were over represented compared with their representation in the general population of the United States (about 14% as of 2017). This may have influenced the genetic analysis. However, it is appreciated that African Americans as a group comprise an incompletely admixed population, with shared African, European, Hispanic, or American Indian ancestry. The fraction of genetic admixture differs significantly from individual to individual. Therefore, certain SNPs and haplotypes may fail to be in HWE from self‐reported African American participants. Such as the rs2227631 SNP in African American of aSAH in this study (31 GG, 27 AG, and 1 AA, HWE *p* = 0.07) and rs1799889 (32 5G5G, 27 5G4G, and 0 4G4G, HWE *p* = 0.02). However, this violation of HWE is relatively limited in terms of its effect on haplotype frequency estimation and individual haplotype prediction (Fallin & Schork, 2000). Small sample size and low statistical power are also limitations to the current study since larger sample sizes are preferred for association studies. Therefore, the findings of current study should be interpreted with caution until larger data sets are available for analysis. Another limitation is the determination of clinical vasospasm which involved using various neurological examinations and corroborating images for diagnosis, and therefore may be vulnerable to subjective opinion.

## 5 CONCLUSION

Taken together, the results suggest an important regulatory role of the *SERPINE1* gene in cascading downstream signals activated by tPA in clinical outcomes of aSAH. The polymorphisms of *SERPINE1* gene could impact the clinical outcomes of aSAH patients. Patients with aSAH who were also G5GGGT homozygotes for the *SERPINE1* gene are at higher risk for DCI and CV, which may increase the mortality and morbidity after aSAH. More research should be carried to confirm the regulatory role of PAI‐1 in aSAH.

## 6 AUTHORS’ CONTRIBUTORS

CG, RS, RT, MS, PF, NV, BW, MH, PH, JP, MM, and RL conducted the design of study, medical procedures, patient care, and enroll subjects to the study. ML conducted the genotyping, genetic statistical analyses, and wrote the manuscript. RL also contributed to the critical revise of the manuscript.

## 7 ETHICS APPROVAL

This study was carried out in accordance with the recommendations of the Institutional Review Boards of the University of Alabama at Birmingham and INOVA Health System with written informed consent from all subjects. All subjects gave written informed consent in accordance with the Declaration of Helsinki. The protocol was approved by the Institutional Review Boards of the University of Alabama at Birmingham and INOVA Health System**.**

## 8 PATIENT CONSENT

Obtained.

## 9 DATA SHARING STATEMENT

Data are available upon reasonable request to the corresponding author.

## CONFLICT OF INTEREST

The authors declare that the research was conducted in the absence of any commercial or financial relationships that could be construed as a potential conflict of interest.
