# Supplementary material for: Haplotype analysis of SERPINE1 gene: Risk for aneurysmal subarachnoid hemorrhage and clinical outcomes
Source: Mol Genet Genomic Med. 2019 Jul 3;7(8):e737. doi: 10.1002/mgg3.737 (PMC6687628; doi:10.1002/mgg3.737)
Supplement: Supplementary file 1 [file MGG3-7-e737-s001.docx]

**Supplemental Tables**

**Supplemental Table 1. Haplotype analysis for Block1 (rs2227631-rs1799889)**

| **Block1 (rs2227631-rs1799889)** | | | | | | | | |
| --- | --- | --- | --- | --- | --- | --- | --- | --- |
| Haplotype (Frequency) | | |  | | Permutation P- value | | | |
| **aSAH** | Case | Control | P (χ^2^) | | Dominant | Additive | | Recessive |
| A4 (0.44) | 120 | 51 | 0.02 | | 0.85 | 0.75 | | 0.76 |
| G5 (0.50) | 157 | 38 |  | | 0.17 | 0.36 | | 0.97 |
| Global |  |  |  | | 0.38 | 0.78 | | 0.94 |
| **DCI** | DCI | No DCI | P (χ^2^) | | Dominant | Additive | | Recessive |
| A4(0.41) | 29 | 89 | 0.41 | | 0.33 | 0.85 | | 0.17 |
| G5(0.53) | 31 | 121 |  | | 0.49 | 0.62 | | 0.12 |
| Global |  |  |  | | 0.38 | 0.46 | | 0.07 |
| **CV** | CV | No CV | P (χ^2^) | | Dominant | Additive | | Recessive |
| A4(0.41) | 29 | 91 | 0.64 | | 0.25 | 0.80 | | 0.36 |
| G5(0.53) | 34 | 122 |  | | 0.81 | 0.99 | | 0.80 |
| Global |  |  |  | | 0.45 | 0.92 | | 0.61 |
| **Aneurysm Location** | Anterior | posterior | P (χ^2^) | | Dominant | Additive | | Recessive |
| A4(0.41) | 99 | 21 | 0.45 | | 0.68 | 0.72 | | 0.94 |
| G5(0.53) | 123 | 33 |  | | 0.68 | 0.45 | | 0.48 |
| Global |  |  |  | | 0.87 | 0.63 | | 0.77 |
| **Hypertension (>140/90)** | Yes | NO | P (χ^2^) | | Dominant | Additive | | Recessive |
| A4(0.41) | 80 | 40 | 0.20 | | 0.87 | 0.45 | | 0.25 |
| G5(0.53) | 115 | 41 |  | | 0.39 | 0.39 | | 0.61 |
| Global |  |  |  | | 0.70 | 0.70 | | 0.49 |
| **Cerebral Edema** | Yes | No | P (χ^2^) | | Dominant | Additive | | Recessive |
| A4(0.41) | 36 | 83 | 0.02 | | 0.41 | 0.17 | | 0.17 |
| G5(0.53) | 28 | 125 |  | | 0.53 | 0.20 | | 0.15 |
| Global |  |  |  | | 0.65 | 0.38 | | 0.19 |
|  | Permutation P value | | | | | | | |
| **Aneurysm size** | Dominant | | | Additive | | | Recessive | |
| A4(0.41) | 0.62 | | | 0.69 | | | 0.94 | |
| G5(0.53) | 0.64 | | | 0.62 | | | 0.77 | |
| Global | 0.83 | | | 0.88 | | | 0.97 | |
| **Hutt and Hess scale** | Dominant | | | Additive | | | Recessive | |
| A4(0.41) | 0.33 | | | 0.31 | | | 0.60 | |
| G5(0.53) | 0.99 | | | 0.89 | | | 0.82 | |
| Global | 0.62 | | | 0.09 | | | 0.83 | |
| **Fisher CT Scale** | Dominant | | | Additive | | | Recessive | |
| A4(0.41) | 0.67 | | | 0.62 | | | 0.73 | |
| G5(0.53) | 0.56 | | | 0.82 | | | 0.84 | |
| Global | 0.81 | | | 0.83 | | | 0.91 | |
| **Hospital Stay** | Dominant | | | Additive | | | Recessive | |
| A4(0.41) | 0.38 | | | 0.83 | | | 0.44 | |
| G5(0.53) | 0.79 | | | 0.84 | | | 0.59 | |
| Global | 0.61 | | | 0.97 | | | 0.60 | |
| **ICU Stay** | Dominant | | | Additive | | | Recessive | |
| A4(0.41) | 0.69 | | | 0.96 | | | 0.66 | |
| G5(0.53) | 0.90 | | | 0.90 | | | 0.75 | |
| Global | 0.89 | | | 0.98 | | | 0.84 | |
| **Last F/U MRS** | Dominant | | | Additive | | | Recessive | |
| A4(0.41) | 0.20 | | | 0.20 | | | 0.56 | |
| G5(0.53) | 0.40 | | | 0.32 | | | 0.47 | |
| Global | 0.38 | | | 0.44 | | | 0.69 | |
| **Glasgow Coma Scale** | Dominant | | | Additive | | | Recessive | |
| A4(0.41) | 0.89 | | | 0.27 | | | 0.50 | |
| G5(0.53) | 0.32 | | | 0.87 | | | 0.72 | |
| Global | 0.59 | | | 0.05 | | | 0.72 | |

**Supplemental Table 2. Haplotype analysis for Block2 (rs6092-rs6090-rs2227684-rs7242)**

| **Block2 (rs6092-rs6090-rs2227684-rs7242)** | | | | | | | | |
| --- | --- | --- | --- | --- | --- | --- | --- | --- |
| Haplotype (Frequency) | | |  | | Permutation P- value | | | |
| **aSAH** | Case | Control | P (χ^2^) | | Dominant | Additive | | Recessive |
| AGGT(0.05) | 19 | 3 | 0.25 | | 0.07 | 0.06 | |  |
| GGAG(0.45) | 126 | 50 |  | | 0.41 | 0.29 | | 0.37 |
| GGGT(0.46) | 136 | 42 |  | | 0.93 | 0.91 | | 0.94 |
| Global |  |  |  | | 0.32 | 0.24 | | 0.63 |
| **DCI** | DCI | No DCI | P (χ^2^) | | Dominant | Additive | | Recessive |
| AGGT(0.06) | 3 | 16 | 0.47 | | 0.37 | 0.38 | |  |
| GGAG(0.42) | 25 | 99 |  | | 0.42 | 0.63 | | 0.94 |
| GGGT(0.47) | 33 | 97 |  | | 0.46 | 0.25 | | 0.51 |
| Global |  |  |  | | 0.59 | 0.58 | | 0.80 |
| **CV** | CV | No CV | P (χ^2^) | | Dominant | Additive | | Recessive |
| AGGT(0.06) | 4 | 15 | 0.90 | | 0.72 | 0.72 | |  |
| GGAG(0.42) | 32 | 99 |  | | 0.58 | 0.44 | | 0.93 |
| GGGT(0.47) | 35 | 101 |  | | 0.46 | 0.60 | | 0.51 |
| Global |  |  |  | | 0.90 | 0.82 | | 0.80 |
| **Aneurysm Location** | Anterior | posterior | P (χ^2^) | | Dominant | Additive | | Recessive |
| AGGT(0.06) | 15 | 4 | 0.69 | | 0.55 | 0.56 | |  |
| GGAG(0.42) | 110 | 23 |  | | 0.40 | 0.82 | | 0.52 |
| GGGT(0.47) | 107 | 29 |  | | 0.64 | 0.61 | | 0.20 |
| Global |  |  |  | | 0.71 | 0.49 | | 0.27 |
| **Hypertension (>140/90)** | Yes | NO | P (χ^2^) | | Dominant | Additive | | Recessive |
| AGGT(0.06) | 13 | 6 | 0.44 | | 0.98 | 0.98 | |  |
| GGAG(0.42) | 99 | 34 |  | | 0.02(score = 2.36) | 0.15 | | 0.83 |
| GGGT(0.47) | 91 | 44 |  | | 0.90 | 0.12 | | 0.01(score = -2.48) |
| Global |  |  |  | | 0.09 | 0.48 | | 0.03 |
| **Cerebral Edema** | Yes | No | P (χ^2^) | | Dominant | Additive | | Recessive |
| AGGT(0.06) | 6 | 13 | 0.41 | | 0.70 | 0.70 | |  |
| GGAG(0.42) | 27 | 104 |  | | 0.06 | 0.16 | | 0.77 |
| GGGT(0.47) | 35 | 99 |  | | 0.36 | 0.20 | | 0.24 |
| Global |  |  |  | | 0.30 | 0.56 | | 0.52 |
|  | Permutation P value | | | | | | | |
| **Aneurysm size** | Dominant | | | Additive | | | Recessive | |
| AGGT(0.06) | 0.48 | | | 0.47 | | |  | |
| GGAG(0.42) | 0.13 | | | 0.10 | | | 0.28 | |
| GGGT(0.47) | 0.39 | | | 0.23 | | | 0.31 | |
| Global | 0.46 | | | 0.42 | | | 0.41 | |
| **Hutt and Hess scale** | Dominant | | | Additive | | | Recessive | |
| AGGT(0.06) | 0.65 | | | 0.65 | | |  | |
| GGAG(0.42) | 0.24 | | | 0.11 | | | 0.15 | |
| GGGT(0.47) | 0.19 | | | 0.19 | | | 0.49 | |
| Global | 0.48 | | | 0.30 | | | 0.34 | |
| **Fisher CT Scale** | Dominant | | | Additive | | | Recessive | |
| AGGT(0.06) | 0.34 | | | 0.35 | | |  | |
| GGAG(0.42) | 0.05 (score = -2.00) | | | 0.16 | | | 0.89 | |
| GGGT(0.47) | 0.18 | | | 0.02(score = 2.34) | | | 0.01(score = 2.45) | |
| Global | 0.12 | | | 0.14 | | | 0.05 | |
| **Hospital Stay** | Dominant | | | Additive | | | Recessive | |
| AGGT(0.06) | 0.30 | | | 0.30 | | |  | |
| GGAG(0.42) | 0.67 | | | 0.35 | | | 0.25 | |
| GGGT(0.47) | 0.22 | | | 0.15 | | | 0.30 | |
| Global | 0.51 | | | 0.43 | | | 0.39 | |
| **ICU Stay** | Dominant | | | Additive | | | Recessive | |
| AGGT(0.06) | 0.66 | | | 0.66 | | |  | |
| GGAG(0.42) | 0.39 | | | 0.23 | | | 0.25 | |
| GGGT(0.47) | 0.30 | | | 0.15 | | | 0.21 | |
| Global | 0.65 | | | 0.53 | | | 0.31 | |
| **Last F/U MRS** | Dominant | | | Additive | | | Recessive | |
| AGGT(0.06) | 0.22 | | | 0.23 | | |  | |
| GGAG(0.42) | 0.04 (score = -2.01) | | | 0.006 (score = -2.69) | | | 0.02 (score = -2.40) | |
| GGGT(0.47) | 0.01 (score = 2.54) | | | 0.02 (score = 2.38) | | | 0.26 | |
| Global | 0.03 | | | 0.04 | | | 0.05 | |
| **Glasgow Coma Scale** | Dominant | | | Additive | | | Recessive | |
| AGGT(0.06) | 0.84 | | | 0.83 | | |  | |
| GGAG(0.42) | 0.10 | | | 0.05(score = 2.00) | | | 0.11 | |
| GGGT(0.47) | 0.12 | | | 0.06 (score = -1.90) | | | 0.14 | |
| Global | 0.27 | | | 0.24 | | | 0.16 | |

**Supplemental Table 3. Haplotype analysis for Block1 + Block2 (rs2227631-rs1799889- rs6092-rs6090-rs2227684-rs7242)**

| **Block1 + Block2 (rs2227631-rs1799889- rs6092-rs6090-rs2227684-rs7242)** | | | | | | | | |
| --- | --- | --- | --- | --- | --- | --- | --- | --- |
| Haplotype (Frequency) | | |  | | Permutation P- value | | | |
| **aSAH** | Case | Control | P (χ^2^) | | Dominant | Additive | | Recessive |
| A4GGGT(0.09) | 23 | 7 | 0.38 | | 0.70 | 0.61 | |  |
| G5GGAG(0.12) | 34 | 6 |  | | 0.41 | 0.33 | |  |
| A4GGAG(0.32) | 89 | 41 |  | | 0.27 | 0.16 | | 0.24 |
| G5GGGT(0.32) | 100 | 30 |  | | 0.83 | 0.82 | | 0.42 |
| Global |  |  |  | | 0.75 | 0.50 | | 0.31 |
| **DCI** | DCI | No DCI | P (χ^2^) | | Dominant | Additive | | Recessive |
| A4GGGT(0.10) | 6 | 16 | 0.22 | | 0.85 | 0.85 | |  |
| G5GGAG(0.13) | 3 | 30 |  | | 0.33 | 0.28 | |  |
| A4GGAG(0.29) | 22 | 66 |  | | 0.72 | 0.83 | | 0.36 |
| G5GGGT(0.32) | 25 | 72 |  | | 0.56 | 0.09 | | 0.005(score = 2.75) |
| Global |  |  |  | | 0.85 | 0.27 | | 0.008 |
| **CV** | CV | No CV | P (χ^2^) | | Dominant | Additive | | Recessive |
| A4GGGT(0.10) | 4 | 19 | 0.14 | | 0.33 | 0.29 | |  |
| G5GGAG(0.13) | 3 | 31 |  | | 0.12 | 0.11 | |  |
| A4GGAG(0.29) | 24 | 65 |  | | 0.93 | 0.59 | | 0.31 |
| G5GGGT(0.32) | 26 | 74 |  | | 0.85 | 0.18 | | 0.01(score = 2.54) |
| Global |  |  |  | | 0.34 | 0.21 | | 0.01 |
| **Aneurysm Location** | Anterior | posterior | P (χ^2^) | | Dominant | Additive | | Recessive |
| A4GGGT(0.10) | 18 | 5 | 0.82 | | 0.94 | 0.97 | |  |
| G5GGAG(0.13) | 28 | 6 |  | | 0.62 | 0.77 | |  |
| A4GGAG(0.29) | 73 | 16 |  | | 0.59 | 0.85 | | 0.60 |
| G5GGGT(0.32) | 77 | 23 |  | | 0.42 | 0.22 | | 0.22 |
| Global |  |  |  | | 0.93 | 0.77 | | 0.41 |
| **Hypertension (>140/90)** | Yes | NO | P (χ^2^) | | Dominant | Additive | | Recessive |
| A4GGGT(0.10) | 10 | 13 | 0.02 | | 0.001(score= -3.19) | 0.004(score = -2.80) | |  |
| G5GGAG(0.13) | 27 | 7 |  | | 0.04(score=2.02) | 0.19 | |  |
| A4GGAG(0.29) | 65 | 24 |  | | 0.26 | 0.40 | | 0.98 |
| G5GGGT(0.32) | 73 | 27 |  | | 0.62 | 0.77 | | 0.81 |
| Global |  |  |  | | 0.007 | 0.06 | | 0.97 |
| **Cerebral Edema** | Yes | No | P (χ^2^) | | Dominant | Additive | | Recessive |
| A4GGGT(0.10) | 10 | 13 | 0.003 | | 0.03(score= 2.23) | 0.01(score= 2.50) | |  |
| G5GGAG(0.13) | 1 | 32 |  | | 0.006(score = -2.77) | 0.007(score = -2.68) | |  |
| A4GGAG(0.29) | 24 | 64 |  | | 0.96 | 0.75 | | 0.50 |
| G5GGGT(0.32) | 21 | 77 |  | | 0.82 | 0.70 | | 0.61 |
| Global |  |  |  | | 0.04 | 0.02 | | 0.73 |
|  | Permutation P value | | | | | | | |
| **Aneurysm size** | Dominant | | | Additive | | | Recessive | |
| A4GGGT(0.10) | 0.95 | | | 0.89 | | |  | |
| G5GGAG(0.13) | 0.35 | | | 0.32 | | |  | |
| A4GGAG(0.29) | 0.29 | | | 0.34 | | | 0.78 | |
| G5GGGT(0.32) | 0.41 | | | 0.21 | | | 0.16 | |
| Global | 0.62 | | | 0.66 | | | 0.37 | |
| **Hutt and Hess scale** | Dominant | | | Additive | | | Recessive | |
| A4GGGT(0.10) | 0.56 | | | 0.52 | | |  | |
| G5GGAG(0.13) | 0.01(score = -2.66) | | | 0.02(score =-2.47) | | |  | |
| A4GGAG(0.29) | 0.73 | | | 0.78 | | | 0.99 | |
| G5GGGT(0.32) | 0.58 | | | 0.30 | | | 0.20 | |
| Global | 0.05 | | | 0.06 | | | 0.42 | |
| **Fisher CT Scale** | Dominant | | | Additive | | | Recessive | |
| A4GGGT(0.10) | 0.34 | | | 0.40 | | |  | |
| G5GGAG(0.13) | 0.26 | | | 0.28 | | |  | |
| A4GGAG(0.29) | 0.31 | | | 0.41 | | | 0.89 | |
| G5GGGT(0.32) | 0.09 | | | 0.05 | | | 0.20 | |
| Global | 0.36 | | | 0.29 | | | 0.43 | |
| **Hospital Stay** | Dominant | | | Additive | | | Recessive | |
| A4GGGT(0.10) | 0.80 | | | 0.66 | | |  | |
| G5GGAG(0.13) | 0.15 | | | 0.13 | | |  | |
| A4GGAG(0.29) | 0.76 | | | 0.94 | | | 0.72 | |
| G5GGGT(0.32) | 0.35 | | | 0.05(score = 1.91) | | | 0.02(score = 2.53) | |
| Global | 0.55 | | | 0.25 | | | 0.05 | |
| **ICU Stay** | Dominant | | | Additive | | | Recessive | |
| A4GGGT(0.10) | 0.83 | | | 0.70 | | |  | |
| G5GGAG(0.13) | 0.04(score = -1.98) | | | 0.04(score = -1.96) | | |  | |
| A4GGAG(0.29) | 0.78 | | | 0.86 | | | 0.93 | |
| G5GGGT(0.32) | 0.38 | | | 0.06(score = 1.93) | | | 0.01(score = 2.64) | |
| Global | 0.24 | | | 0.11 | | | 0.03 | |
| **Last F/U MRS** | Dominant | | | Additive | | | Recessive | |
| A4GGGT(0.10) | 0.48 | | | 0.27 | | |  | |
| G5GGAG(0.13) | 0.07(score = -1.80) | | | 0.06(score = -1.89) | | |  | |
| A4GGAG(0.29) | 0.09(score = -1.66) | | | 0.07(score = -1.83) | | | 0.26 | |
| G5GGGT(0.32) | 0.16 | | | 0.04(score = 2.05) | | | 0.04(score = 2.11) | |
| Global | 0.15 | | | 0.09 | | | 0.08 | |
| **Glasgow Coma Scale** | Dominant | | | Additive | | | Recessive | |
| A4GGGT(0.10) | 0.98 | | | 0.92 | | |  | |
| G5GGAG(0.13) | 0.002(score = 2.97) | | | 0.002(score = 3.030) | | |  | |
| A4GGAG(0.29) | 0.56 | | | 0.72 | | | 0.84 | |
| G5GGGT(0.32) | 0.41 | | | 0.18 | | | 0.13 | |
| Global | 0.03 | | | 0.02 | | | 0.31 | |

**F/U: Follow up**

**Supplemental Table 4**

**Further investigation on rs7242**

For rs7242 SNP in the 3' untranslated region, we further investigated the factors that may cause an allele-specific impact on *SERPINE1* gene expression. The first is the putative micro RNAs (miRNA) in *Homo sapiens* that could have allele-specific binding to the seed region. We used bioinformatic tool MicroSNiPer^26^ for this investigation. The results are shown in Table 4. The results showed that there are more possible miRNA binding sites for the G allele than for the T allele. Computationally, there were four miRNAs influenced by the G allele and two miRNAs influenced by the T allele in the seed region. Moreover, the hsa-mir-4256 miRNA was suggested to have differential binding for the two alleles, 9bp seed length for the G allele versus 7bp seed length for the T allele. We further investigated the RNA binding protein that may be allele-specific for rs7242. By mapping binding motifs of RNA binding proteins in RBPmap for rs7242, the hydroquinone (NADH) oxidase ENOX1(also known as Ecto-NOX disulfide-thiol exchanger 1), binds to AAGAAA[G] motif for rs7242 G allele, but does not bind to this motif when the T allele substitutes for the G allele.

**Table 4. Putative miRNA binding with alleles of rs7242**

| rs7242(+ strain), NCBI Reference Sequence: NM_000602 | | | |
| --- | --- | --- | --- |
|  | miRNA | Seed Length | Binding sequence |
| T allele | hsa-mir-6719-3p | 8bp | 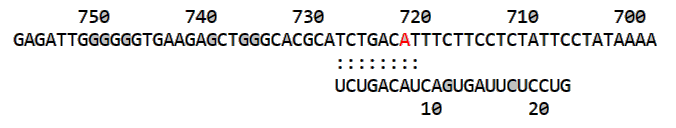 |
|  | hsa-mir-4256 | 7bp | 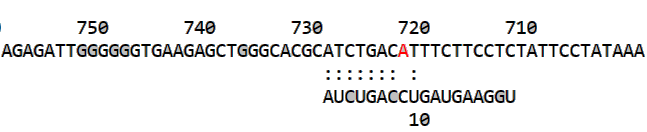 |
| G allele | hsa-mir-4256 | 9bp | 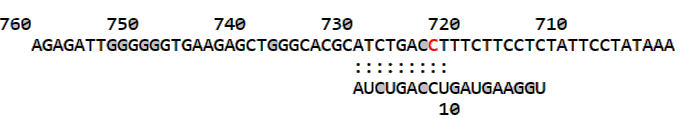 |
|  | hsa-mir-192-5p | 7bp | 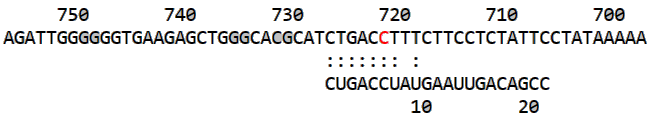 |
|  | hsa-mir-215 | 6bp | 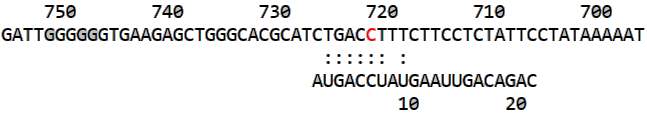 |
|  | hsa-mir-3661 | 6bp | 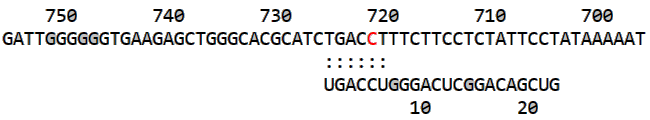 |
